# An analysis of whether a working-age ward-based liaison psychiatry service requires the input of a liaison psychiatrist

**DOI:** 10.1192/pb.bp.115.052837

**Published:** 2017-06

**Authors:** Elspeth A. Guthrie, Aaron T. McMeekin, Sylvia Khan, Sally Makin, Ben Shaw, Damien Longson

**Affiliations:** 1 University of Manchester; 2 Manchester Royal Infirmary; 3 Parkwood Hospital, Blackpool; 4 Birch Hill Hospital, Rochdale; 5 Royal Bolton Hospital, Bolton; 6 Manchester Mental Health and Social Care Trust

## Abstract

**Aims and method** This article presents a 12-month case series to determine the fraction of ward referrals of adults of working age who needed a liaison psychiatrist in a busy tertiary referral teaching hospital.

**Results** The service received 344 referrals resulting in 1259 face-to-face contacts. Depression accounted for the most face-to-face contacts. We deemed the involvement of a liaison psychiatrist necessary in 241 (70.1%) referrals, with medication management as the most common reason.

**Clinical implications** A substantial amount of liaison ward work involves the treatment and management of severe and complex mental health problems. Our analysis suggests that in the majority of cases the input of a liaison psychiatrist is required.

Liaison psychiatry services in England have recently seen considerable expansion, following the positive evaluation of the Rapid Assessment Interface and Discharge Team (RAID) service in Birmingham.^1^ The independent evaluation suggested that this well-staffed multidisciplinary liaison psychiatry service returned considerable cost savings for people with mental health problems in that general hospital and those attending the emergency department.

The 2015 annual survey of liaison psychiatry in England demonstrated that many services are too poorly resourced to be delivering benefits to patients as envisaged by the RAID study,^2^ while the recent *Guidance on Developing Models for Liaison Psychiatry Services* has shown great variability in service provision.^3^ The relative lack of consultant psychiatric input suggests that the specialist skills offered by consultant liaison psychiatrists may be poorly understood. To examine the multidisciplinary liaison psychiatry team, we aimed to determine the proportion of referrals to a ward-based liaison service which required the involvement of a psychiatrist in a busy tertiary teaching hospital in England. This project was carried out as part of a registered audit (Audit Project 2125) with Manchester Mental Health and Social Care Trust.

There are many descriptions of the activity of liaison services, but we believe this is the first time the role of a psychiatrist has been addressed in depth. We hope our findings will help inform decisions regarding staffing and the configuration of multidisciplinary liaison psychiatry teams. We focused on a ward-based liaison service for adults of working age.

## Method

We carried out a case series of the electronic records of consecutive referrals to a ward-based liaison service at a large teaching hospital in Manchester, over a 12-month period from 18 June 2013 to 17 June 2014. The hospital has approximately 750 beds, with a large renal and critical care unit. There is also a maternity unit on site, with over 8500 deliveries per annum, and an eye hospital. Our liaison service reviews referrals covering an age range of 16–65 by the next working day. Routine alcohol referrals, covered by two specialist nurses, may also be received by our service.

The following details were recorded for each referral: age, gender, number of face-to-face contacts carried out by the team, amount of time the team had ongoing contact with the patient (days), reason for referral, referrer type, psychiatric diagnosis using clinical judgement according to ICD-10 criteria,^4^ employment of the Mental Health Act 1983, and disposal.

### Involvement of a liaison psychiatrist

To determine the proportion of patients who required the input of a psychiatrist, we developed criteria for contacts or interventions that required medical input and could not be undertaken by a non-psychiatrist. We deemed the involvement of a psychiatrist would be necessary in any of the following scenarios: Mental Health Act employed, rescinded or considered; medication management; treatment with psychotropic medications commenced in the general hospital; complex diagnostic matters; behavioural disturbance in physically unwell patients where advice about appropriate sedation is required; complex capacity assessments; and a specific request for a consultant psychiatrist opinion. Each case was reviewed and rated by a member of the team. Box 1 provides detailed criteria for each of these scenarios. We recognise that there are many other kinds of interventions a psychiatrist may carry out as part of liaison work, but we wished to focus solely on interventions which could not be carried out by someone other than a psychiatrist. Many of the interventions therefore require some kind of expertise involving medical knowledge or prescribing of psychotropic medication in patients who are physically unwell.

Summary scores for normally distributed data are presented as means and standard deviations (s.d.). The activity data were not normally distributed, so summary scores are presented in the form of medians and interquartile ranges (IQR). Comparisons of continuous activity data were carried out using non-parametric statistical tests. Mann-Whitney *U*-tests were used to compare continuous data. As in some cases we had few activity data, with only one or two patients in each group, groups with very small sizes (two patients or fewer) were not included in the statistical analyses.

## Results

The service received 344 referrals over the study period and carried out 1259 face-to-face contacts with patients. The average number of ‘face-to-face’ contacts per patient was 3.7 (s.d. = 5, range 1–33). The median age of patients seen was 48 years (IQR 36–59), and 184 (53.4%) were female. At any one time, between 8 and 15 patients were under review by the team. The majority of referrals were from physicians (all medical departments) (*n* = 227, 66.0%), 59 (17.2%) were from surgery, 31 (9%) from the maternity hospital and 23 (6.7%) from the critical care unit. One referral came from the eye hospital and a further 3 were from the liaison service for older adults, but these patients were best managed by the adults of working age team.

Table DS1 in the online supplement provides a snapshot of the patients under the care of the service on one day in May 2014, and is typical of the kind of patients who are under review by the service at any given time. Five patients had severe mental illness (four had schizophrenia and one had bipolar disorder). Two had been treated under the Mental Health Act and there was a possibility that one more person may have needed treatment. One person was on a community treatment order (CTO). Four patients had voiced recent suicidal ideas, two had complex organic mental disorders, and three had psychological reactions to physical illness or difficulties with their behaviour in an acute hospital setting.

### Referrals which required the involvement of a psychiatrist

Table 1 illustrates the number of referrals which needed a psychiatrist. Of the 344 referred patients who saw a psychiatrist, we deemed on the basis of our criteria that a psychiatrist was required for 241 patients (70.1%). Patients who required a psychiatrist needed more face-to-face contacts than those who did not require a psychiatrist (median 2 (IQR 1–5) *v*. median 1 (IQR 1–2)), and were under the liaison service for a greater period of time (median 7 days (IQR 1–14) *v*. median 1 day (IQR 1–7)).

**Table 1:** Referrals and service workload depending on whether patient required a psychiatrist or not

		Face-to-face contacts		Days in contact with service, median (IQR)
Type of clinical problem	Referrals, *n* (%)	*n* (%)	Median (IQR)	
Requires psychiatrist	241 (70.1)	1039 (82.5)	2 (1–5)***	7 (1–14)***

Does not require psychiatrist	103 (29.9)	220 (17.5)	1 (1–2)	1 (1–7)

Total	344	1259	2 (1–5)	4 (1–13)

IQR, interquartile range.

*** *P*< 0.001 (requires *v.* does not require a psychiatrist).

According to our criteria, the most common reason for psychiatric input was medication management (Table 2): 77 patients (32.0%) required input regarding their current psychotropic medication use. Of interest, 10 of these patients were referred for clozapine management which required over 100 face-to-face contacts from the team. 56 patients were started on treatment for their mental health problems while they were in hospital (i.e. patient started on psychotropic medication). There were complex diagnostic issues in 38 patients and the Mental Health Act was considered in 23 patients, but only actually implemented in 18. In 14 cases the patient required a complex capacity assessment, 10 patients presented with challenging behaviour requiring advice about sedation, and a specific consultant opinion or involvement was requested in 6 patients. The categories are not mutually exclusive.

**Table 2:** The number of patients who required a psychiatrist according to the categories in the study

Clinical categories	*n*	Percentage of total requiring psychiatrist
Medication management	77	32.0%

Treatment with psychotropic drugs	56	23.2%

Complex diagnostic issues	38	15.8%

Mental Health Act	23	9.5%

Management of severe mental illness	17	7.1%

Complex capacity assessments	14	5.8%

Management of behavioural disturbance	10	4.1%

Specific liaison consultant review	6	2.5%

**Box 1** Definitions of categories used to determine whether the involvement of a liaison psychiatrist was required

- Mental Health Act: situations where the Mental Health Act has been applied or its potential use has been seriously considered
- Medication management: consultation in which there was a specific issue about psychotropic medication the patient was taking due to a change in their physical health. This may involve stopping, switching or another action.
- Management of behavioural disturbance in the general hospital: involving advice about medication, where a psychiatrist has provided assessment, advice and guidance about using sedating medication. The psychiatrist will have considered the patient's underlying physical health problems (e.g. renal failure) in making the decision regarding type and dosage of medication. Other non-pharmacological aspects for management of acute behavioural disturbance in physically unwell patients are not considered here, as they are not exclusively carried out by liaison psychiatrists
- Complex diagnostic matters: diagnosis or understanding of a clinical problem which required knowledge of specific medical disorders
- Capacity: requests for medically complex capacity assessments, where the patient has a history of a psychiatric condition which may be interfering with their judgement to give informed consent to potentially life-saving treatment
- Treatment with psychotropic medications commenced in the general hospital: treatment with a psychotropic agent that was commenced in the general hospital by the liaison team, in a patient who had ongoing physical health concerns
- Specific request for consultant liaison psychiatrist opinion: the referring consultant specifically requested a consultant psychiatric opinion or involvement

**Box 2** Categories of clinical scenarios where we judged a psychiatrist was required (examples)

- **Mental HealthAct**: Female (age range 50–60) with diagnosis of schizophrenia. Admitted with a ruptured oesophagus. Clozapine had been stopped prior to admission as she had refused to take it. Her psychosis had relapsed and she was floridly psychotic in hospital. Following repair of her oesophagus she required 6–8 weeks bed rest for the repair to heal. She was treated under Section 3 of the MHA with covert medication (clozapine). Her mental state returned to normal. She had a good physical and mental health recovery. She later agreed to continue to take clozapine on a voluntary basis.
- **Medication management**: Female (age range 30–40) admitted following collapse and found to have very low sodium. Diagnosis of schizophrenia. In discussion with medical team, all psychotropic medication was stopped. Haloperidol started cautiously. Usual medications re-started after physical recovery. Discharged to CMHT.
- **Management of behavioural disturbance**: Male (age range 40–50) who was admitted with delirium, barricaded himself and 3 other patients in a 4-bedded side room. History of hydrocephalus and other abnormal neurological signs. Input required sedation to manage the current situation and to facilitate medical investigations including brain MRI.
- **Diagnosis**: Male (age range 50–60) admitted from nursing home with a history of severe weight loss. History of schizophrenia and extrapyramidal side-effects attributed to neuroleptics. Huntington's chorea diagnosed by consultant psychiatrist.
- **Capacity**: Male (age range 50–60) with history of schizophrenia. Jumped off a bridge when 22 years of age, paraplegic following this. Psychosis treated well for years on clozapine. Developed bowel obstruction, multi-organ failure. Clozapine stopped. On regular haemodialysis. Chronically psychotic. Refusing dialysis. Complicating factors, low mood, chronic psychosis (at times he believes he is Christ and can be resurrected).
- **Treatment with psychotropic drugs commenced in the general hospital**: Male (age 60–70) admitted after stabbing his wife in the back and then stabbing himself 4 times in the abdomen. Diagnosed with depressive disorder. Treatment started with antidepressants while receiving medical treatment on ward. Mood improved.
- **Specific request for consultant liaison psychiatric opinion**: Male (age range 40–50) with a history of gastric problems and feeding difficulties. Had had gastrectomy and had been started on TPN. Had been in hospital for over a year. Staff suspected that reliance on TPN was far more than clinically indicated but all efforts to reduce it failed. Patient was aggressive on occasions with staff, made frequent complaints about staff and at times threatened self-harm. Consultant opinion was specifically sought regarding the risks of moving to home with TPN feeding.

CMHT, community mental health team; MHA, Mental Health Act; MRI, magnetic resonance imaging; TPN, total parenteral nutrition.

Table 2 shows the number of patients who required a psychiatrist according to the categories developed for this study.

Clinical illustrations of actions or interventions which were judged to require the involvement of a psychiatrist, according to each category, are provided in Box 2.

Table 3 shows the number and percentage of patients who required input from a liaison psychiatrist according to the most common psychiatric diagnoses. Patients with bipolar affective disorder, schizophrenia, Korsakoff's psychosis, amnesic syndrome and somatoform disorder required the involvement of a psychiatrist in over 80% of all cases, whereas for patients with anxiety/panic disorder, adjustment disorder or dementia the requirement was much lower.

**Table 3:** Patients who required input from a liaison psychiatrist according to diagnosis

Psychiatric diagnosis	Required psychiatrist (% of total seen)
Somatoform disorders	9 (100.0%)

Bipolar affective disorder	23 (92.0%)

Amnesic syndrome	11 (91.7%)

Korsakoff's psychosis	11 (91.7%)

Schizophrenia	42 (82.4%)

Depression	100 (74.1%)

Miscellaneous including eating disorders, intellectual disability	5 (71.4%)

Personality disorder	9 (69%)

Substance misuse	11 (68%)

Delirium	15 (65.2%)

Anxiety/panic disorder	6 (50.0%)

Dementia	2 (33.3%)

No diagnosis	8 (32.0%)

Adjustment disorder	2 (20.0%)

## Discussion

Our findings suggest that a ward-based liaison psychiatry service for working-age adults in a large teaching hospital requires the input of liaison psychiatrists. We deemed that a psychiatrist was essential in the assessment or management of approximately 70% of all referrals to the service, whereas approximately 30% could be reviewed by other members of a liaison team. We based this judgement on clear, definable actions or aspects of care that necessitated the involvement of a psychiatrist. A consultant liaison psychiatrist would of course have many other roles, but for the purposes of this study we limited our focus to interventions or actions where the role of a psychiatrist was unequivocal.

Many of the patients seen by the service had complex physical and mental health needs. Table DS1 provides a snapshot of the work, and illustrates that it is necessary to involve a psychiatrist in the management of a large proportion of referrals. Out of the 12 patients under the care of the team on one day in May 2014, 9 required the input of a psychiatrist.

Certain patients with diagnoses such as adjustment disorder and dementia were unlikely to require psychiatric input, whereas high rates of psychiatric involvement were required for patients with severe mental illness and somatoform disorders. Psychiatric input was also needed in the management of patients with Korsakoff's psychosis, because locally a formal diagnosis from a psychiatrist is required in order to access particular kinds of Social Services support.

This study has three major limitations. First, data were based on routine clinical entries made using a National Health Service (NHS) electronic record system. It is possible that this may have led to an underestimation of the numbers of patients requiring psychiatric input due to a lack of recording certain data (e.g. details about psychotropic medication). It is very unlikely that it would have led to an overestimate of our findings. Second, this study was undertaken in a teaching hospital, with a large critical care unit, a large renal unit, a very busy maternity hospital and other specialist centres. It may not reflect the work of a liaison service in a district general hospital, but it emphasises the need to take account of local variations in acute hospital services when planning a liaison service. Third, this liaison service is a ward-based service only. Approximately a third of liaison services run out-patient clinics for complex cases requiring psychiatric time. Clinics can take psychiatrists away from acute ward cover and require different planning and staff resources compared with a ward-based liaison service.

The methods we employed, however, can easily be used by other services to estimate the requirement for input from a liaison psychiatrist, and this is likely to vary depending on the setting and age range of patients seen. As this research team consisted entirely of psychiatrists, we may have overestimated the need for the skills of our own discipline when creating the criteria and applying them. However, we have provided clinical examples to illustrate our decision-making process and thus expose it to critical examination.

Our results suggest that liaison psychiatrists have a pivotal role in ward-based liaison services for adults of working age, and this may be particularly important in a teaching hospital setting. Our work also provides support for the recent commissioning guidance for liaison psychiatry services in England developed by the Department of Health, which suggests that liaison psychiatry services in a teaching hospital/inner city setting may require additional consultant psychiatric input.^7^
